# Central antinociceptive activity of peripherally applied botulinum toxin type A in lab rat model of trigeminal neuralgia

**DOI:** 10.1186/s40064-016-2071-2

**Published:** 2016-04-11

**Authors:** Chuanjie Wu, Nanchang Xie, Yajun Lian, Hongliang Xu, Chen Chen, Yake Zheng, Yuan Chen, Haifeng Zhang

**Affiliations:** Department of Neurology, the First Affiliated Hospital of Zhengzhou University, Zhengzhou, 450052 China

**Keywords:** Trigeminal neuralgia, Botulinum toxin type A, Central antinociceptive activity, Rat

## Abstract

**Background:**

BoNT-A is often used in the clinical treatment for movement disorders. In recent years, various clinical studies suggest that BoNT-A can effectively alleviate pain caused by trigeminal neuralgia (TN); however, its mechanism remains unclear.

**Methods:**

In this study, we used a lab rat model for TN produced by chronic constriction injury of the infraorbital nerve (ION-CCI). Restrained rats were injected subcutaneously with BoNT-A into the whisker pad tissue (ipsilaterally to the nerve injury) 14 days after the ION-CCI. Allodynia was tested by Von Frey filaments and TRPs and cSNAP-25 were tested by western blot.

**Results:**

Peripheral application of BoNT-A (3, 10 U/kg) significantly increased the pain threshold of ION-CCI rats. Rota-rod test showed that BoNT-A administration at doses tested did not significantly affect rat motor coordination. By probing for a specific marker for BoNT-A, cleaved synaptosomal-associated protein 25 (cSNAP-25), we found that peripheral application of BoNT-A (10 U/kg) affected brainstem Vc, which could be blocked by the axonal transport blocker colchicine. In addition, western blot analysis showed that in the Vc region of ION-CCI rats, the expression levels of TRPA1, TRPV1, TRPV2 and TRPM8 increased, whereas peripheral application of BoNT-A significantly lowered the high expression of TRPA1, TRPV1 and TRPV2, but not TRPM8 at 7 days after BoNT-A injection.

**Conclusions:**

The finding of this study suggest that peripherally applied BoNT-A can produce antinociceptive effects in ION-CCI model. The underlying mechanisms may be BoNT-A acts on the Vc via axonal transport, inhibits the high expression of TRPA1, TRPV1 and TRPV2, and reduces central sensitization.

## Background

Trigeminal neuralgia (TN) is episodic facial pain that is usually described to feel like a unilateral electric shock. This neuropathic disorder has been shown to be profoundly distressing and to negatively impact the patient’s well-being (Hall et al. 2006). According to epidemiological studies, approximately 4–28.9/100,000 persons worldwide experience TN (Hall et al. 2006; Dieleman et al. 2008; Katusic et al. 1990). Patients with TN usually present a clinical treatment challenge. The antiepileptic drugs are usually used first in an attempt to treat TN. However, treatment with antiepileptic drugs results in more adverse reactions, and requires daily administration. In addition, long-term use can cause a gradual decline of drug efficacy (Taylor et al. 1981).

Botulinum toxin type A (BoNT-A) is one of the serotypes (A, B, C1, C2, D, E, F and G) of botulinum neurotoxins derived from Clostridium botulinum (Setler 2002). Brin et al. (1987) reported the use of BoNT-A for treatment of dystonia, which results in relief of dystonia symptoms, as well as significant pain experience improvement in 74 % of the patients. Subsequently, the antinociceptive effects of BoNT-A are gradually recognized (Luvisetto et al. 2015). With in-depth understanding, several clinical studies indicate that BoNT-A can effectively alleviate TN (Zuniga et al. 2008; Ngeow and Nair 2010; Bohluli et al. 2011). In 2012, we first used the RCT experimental method to demonstrate that BoNT-A can effectively alleviate the pain caused by TN with mild adverse reactions (Wu et al. 2012). Subsequent studies further confirm the effectiveness of BoNT-A for the treatment of TN (Zhang et al. 2014; Xia et al. 2016; Li et al. 2014). However, the mechanism of BoNT-A treatment for TN remains unclear. Currently, most studies on the mechanism of the antinociceptive effects of botulinum toxin focus on the formalin-induced pain model, as well as pre-application of BoNT-A to explore its role in pain prevention (Cui et al. 2004).

As most case of TN are caused by sensory nerve root compression (Zakrzewska and Linskey 2014), Vos et al. (1994) developed a lab rat model of TN produced by chronic constriction injury of the infraorbital nerve (ION-CCI), which is a branch of the trigeminal nerve. This model reproduces important aspects of TN, including signs of abnormal spontaneous pain-related behavior and mechanical allodynia (Vos et al. 1994).

The aim of the present study is to investigate the antinociceptive effects of BoNT-A in the rat ION-CCI model, and whether BoNT-A exerts antinociceptive function by acting on the central nervous system. In addition, we also examined the potential central antinociceptive mechanisms of BoNT-A.

## Methods

### Animals and trigeminal neuralgia model

Adult male Sprague-Dawley rats (Experimental Animal Center of Zhengzhou University) weighing 220–300 were used. All rats were housed in climate-controlled rooms on a 12/12 light/dark cycle with water and standardized rodent diet available ad libitum. The experimental procedures were approved by the Commission of Zhengzhou University for ethics of experiments on animals in accordance with international standards (No. YFY2015096).

The trigeminal neuralgia model was performed by the method as described previously (Imamura et al. 1997). Animals were anesthetized by a single intraperitoneal injection of 10 % chloral hydrate (0.2 ml/kg). All incisions were made intraorally, which allowed the hair on the snout and vibrissae to remain intact. An incision approximately 10 mm long was made along the gingivobuccal margin. The incision was placed proximal to the first molar. The infraorbital nerve was dissected and two chromic catgut ligatures (4-0) were placed around the nerve spaced 2 mm apart. The ligatures reduced the diameter of the nerve by just a noticeable amount and they did not interrupt the epineural circulation. The incision was sutured at three points using 4.0 silk. The sham operation was identical except that the ION was not ligated.

### Drug administration

BoNT-A (Hengli, Lanzhou, China) was reconstituted in adequate volume of 0.9 % saline. Restrained rats were injected subcutaneously with BoNT-A (30 μl) into the whisker pad tissue (ipsilaterally to the nerve injury) 14 days after the ION-CCI using a Hamilton syringe needle (Hamilton Microliter 801, Hamilton, Bonaduz, Switzerland). The dosed used were 3, and 10 U/kg BoNT-A, respectively. For control rats, 30 μl normal saline was injected.

Colchicine (Saxama, Wuhan, China) was reconstituted in normal saline to obtain the 5 mM concentration. Colchicine or normal saline (2 μl) was injected into the trigeminal ganglion (ipsilaterally to the nerve injury) of anesthetized rat as described previously (Neubert et al. 2005). The Hamilton syringe needle (Hamilton Microliter 801, Hamilton, Bonaduz, Switzerland) was briefly inserted medially (1–2 mm) to the palpated portion of the zygomatic process through the infraorbital foramen. The needle was positioned at a 10 degree angle relative to the midline of the head. The tip of the needle was advanced approximately 20 mm along the infraorbital canal and subsequently through the foramen rotundum then the colchicines or isometric normal saline was injected.

### Mechanical allodynia testing

Von Frey hairs (North coast medical Inc, Morgan Hill, CA) were used for mechanical stimulation. The filaments produced a bending force of 0.008, 0.02, 0.04, 0.07, 0.16, 0.4, 0.6, 1.0, 1.4, 2.0, 4.0, 6.0, 8.0, 10.0, 15.0, 26.0, 60.0, 100, 200 and 300 g. Testing was performed as previously described in detail by Vos et al. (1994). In brief, a single rat is placed in a small transparent plastic cage for 10 min to accommodate to the experimental environment until they assumed their normal sniffing/no locomotion position. Testers were not informed as to which rats had CCI or sham surgery. After the acclimatization sessions the tester was able to apply the von Frey hair to the ipsilateral territory of the injury ION (whisker pad), starting at 0.008 g, until a defined behavioral response was elicited. When rats showed positive reactions, the intensity was recorded, which was the pain threshold. Positive reactions included (1) withdrawal reaction with rapid head withdraw; (2) escape or aggressive behavior, manifested as escape, curling up, hiding its head, or biting or grasping the stimulant; (3) asymmetric face grooming, rat displays an uninterrupted series of at least three face-wash strokes directed to the stimulated facial area. If rats did not show any of the aforementioned reactions at the stimulation intensity of 26 g, then the pain threshold was given 26 g. Measurements were performed three times in ten min intervals and the mean of the measurements is used.

### Rota-rod test

To investigate whether BoNT-A has a systemic effect, we tested the effect of BoNT-A (at dose of 3 and 10 U/kg) on rat’s performance on the Rota-rod test. For the Rota-rod experimental apparatus (Ugo Basile, Italy), the rod diameter was 6 cm, and the rotating speed was set to gradually increase from 5 to 40 r/min in 5 min. The latency for the rat to fall from the rotating rod was recorded. Each rat was subjected to four consecutive tests and results were averaged.

### Western blot

The caudal subnucleus of the spinal trigeminal nucleus (Vc) was collected from deeply anesthetized, rapidly decapitated rats at designated time, either proceeded immediately for biochemical studies or kept at −80 °C until use. Western blot was performed as described previously (Xie et al. 2014). Briefly, 30 μg of protein was separate by SDS-polyacrylamide gel electrophoresis (PAGE) and then transferred to PVDF membranes. After blocking in 5 % fat-free milk in Tris-buffered saline containing 0.1 % Tween, immunoblots were probed with antibodies to cleaved synaptosomal-associated protein 25 (cSNAP-25) (1:2000; GeneTex, USA), transient receptor potential ankyrin 1 (TRPA1) (1:3000; Abcame, UK), transient receptor potential vanilloid type 1 (TRPV1) (1:1000, Sigma, USA), transient receptor potential vanilloid type 2 (TRPV2) (1:1500; Sigma, USA) and transient receptor potential vanilloid melastatin 8 (TRPM8) (1:1000; Abcame, UK). The same blots were stripped and reprobed with antibodies to β-actin (1:5000; Santa Crus, USA). The blots were then incubated with horseradish peroxidase (HRP)-conjugated secondary antibody (1:10,000, Boster, Wuhan, China) for 1 h at 37 °C. Immunoreactivity was visualized by chemiluminescence and exposure to a film. Band intensities were quantified by densitometric analysis using a densitometer.

### Statistical analysis

All data is expressed as mean ± SD. The statistical significance was assessed using One-way ANOVA and the New-man-Keuls test. All statistical analyses were performed using SPSS 18.0. P < 0.05 was considered statistically significant.

## Results

### Antinociceptive effects of peripherally applied BoNT-A

**Fig. 1:**
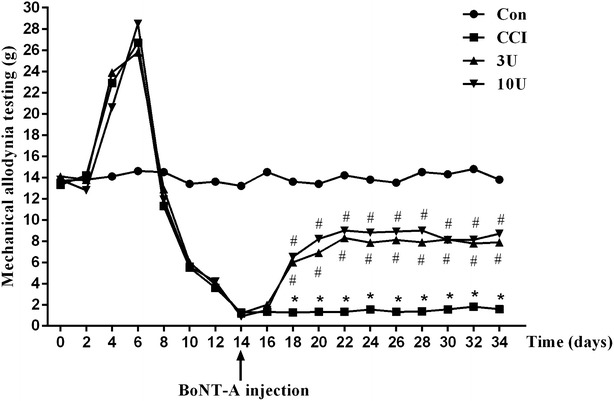
Effect of BoNT-A on mechanical hypersensitivity induced by ION-CCI. Rats underwent either ION-CCI or sham operation. Fourteen days after the operation, BoNT-A (3, 10 U/kg) or normal saline was injected into the whisker pad ipsilateral to the operation site. Measurement were performed on the ipsilateral to the nerve injury. *P < 0.05 versus control group and ^#^P < 0.05 versus CCI group

Von Frey hairs were used to examine the antimociceptive effects of BoNT-A. Our results showed that 14 days after ION-CCI, the ipsilateral pain threshold of the ION-CCI group was significantly reduced compared to the sham-operated group (P < 0.05). BoNT-A (3, 10 U/kg) as injected subcutaneously into the ipsilateral whisker pad tissue 14 days after the ION-CCI. Four days after the subcutaneous injection with BoNT-A, pain thresholds of each BoNT-A dose group significantly increased compared to the control group (P < 0.05). This antimociceptive effects reached a maximum level at 8 days and remained significantly elevated until 20 days. The 10 U/kg group had better antimociceptive effects than the 3 U/kg group; however, the difference between them was not statistically significant (P > 0.05) (Fig. 1).

### Effects of BoNT-A on rat motor coordination ability

BoNT-A (3, 10 U/kg) injected subcutaneously into the ipsilateral whisker pad did not affect the motor coordination ability of rats. Rota-rod test is a commonly used method to reflect the motor coordination ability of rats. Results of the Rota-rod test showed that 7 days after injection, the averaged latencies of rats to fall from the rotating rod of the two dose groups were not statistically significantly different from those of the control group (Table 1).

**Table 1:** Effects of BoBT-A on rat motor coordination ability (accessed by Rota-rod test)

	3 U (s)	10 U (s)
Before BoNT-A treatment	268.2 ± 13.8	271.8 ± 16.6
Seven days after BoNT-A treatment	255.7 ± 8.5*	278.6 ± 11.4*

* P > 0.05 versus Before BoNT-A treatment

### SNAP-25 cleavage in Vc and central antinociceptive effects after BoNT-A peripheral application is axonal transport dependent

**Fig. 2:**
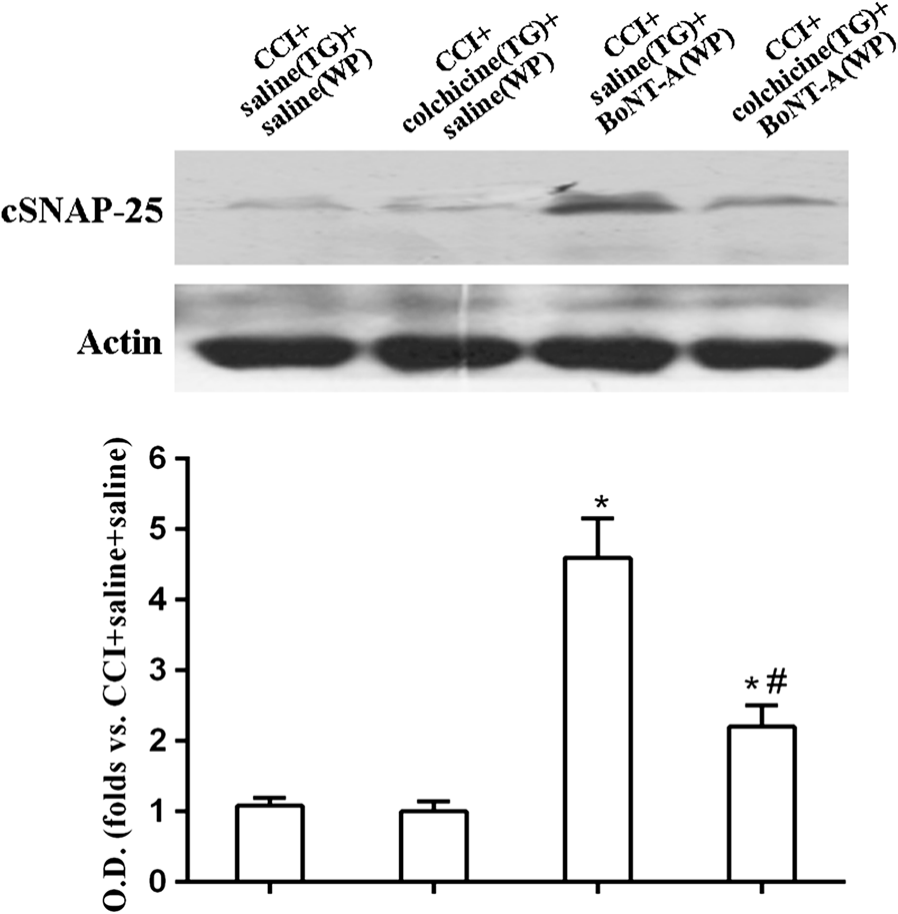
Western blot analysis and quantitative analysis of cSNAP-25 in Vc 7 days after BoNT-A treatment. β-actin was used as an internal standard. Only the representative Western blots of them are illustrated in this figure. Data were mean ± SD. (n = 6/group). TG indicates injection into the trigeminal ganglion; WP indicates injection into the facial whisker pad. *P < 0.05 versus CCI + saline(TG) + saline(WP) group and ^#^P < 0.05 versus CCI + saline(TG) + BoNT-A(WP) group

**Fig. 3:**
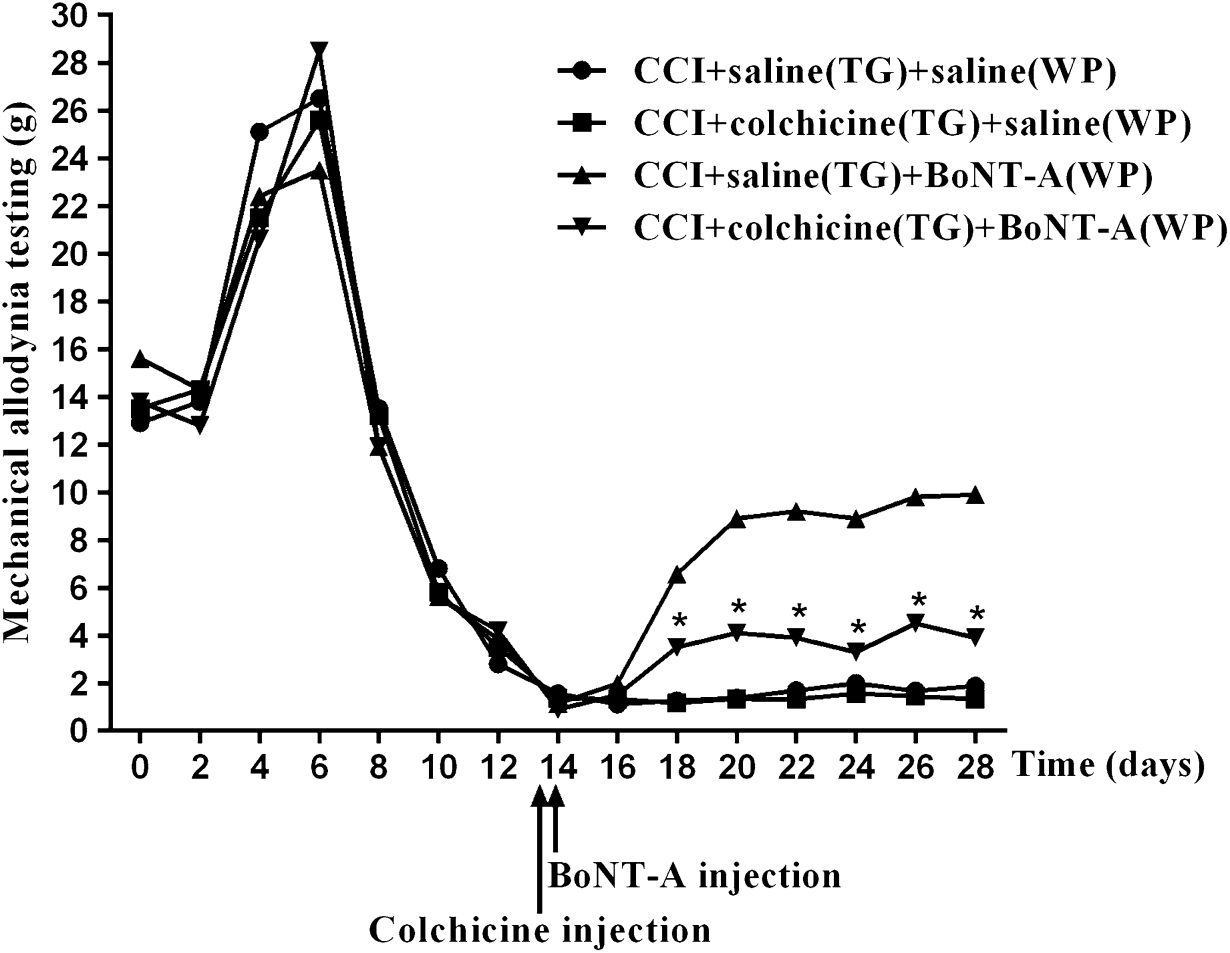
Axonal transport blocker colchicine blocked antinociceptive effects of BoNT-A (10 U/kg). Rats underwent either ION-CCI or sham operation. Injection of BoNT-A (3, 10 U/kg) or normal saline into the whisker pad ipsilateral to the operation site was performed 14 days after the operation. Colchicine or normal saline was injected into ipsilateral trigeminal ganglion 12 h before the application of BoNT-A. TG indicates injection into the trigeminal ganglion; WP indicates injection into the facial whisker pad. *P < 0.05 versus CCI + saline(TG) + BoNT-A(WP) group

Among the other Botulinum toxins, BoNT-A cleaves specific sites of synaptosomal-associated protein 25 (SNAP-25) to inhibit the exocytosis of neurotransmitters from the nerve terminals. So, the cSNAP-25 can be used as a reliable marker of the BoNT-A diffusion and local action. Fourteen days after ION-CCI operation, BoNT-A (10 U/kg) injection was performed. Seven days after BoNT-A injection, the level of cSNAP-25 in the Vc in the BoNT-A treatment group significantly increased compared to control group (P < 0.05). When axonal transport blocker colchicine was injected ipsilateral to BoNT-A injection into the trigeminal ganglion 12 h before the BoNT-A treatment, BoNT-A (10 U/kg) failed to increase the level of cSNAP-25 (P > 0.05) in the Vc (Fig. 2). Behavioral studies also indicated that compared to the control group, ipsilateral colchicine injection into the trigeminal ganglion resulted in disappearance of the antinociceptive effects of BoNT-A (10 U/kg) (P > 0.05) (Fig. 3).

### Effect of BoNT-A on TRPs expression in Vc tissues

**Fig. 4:**
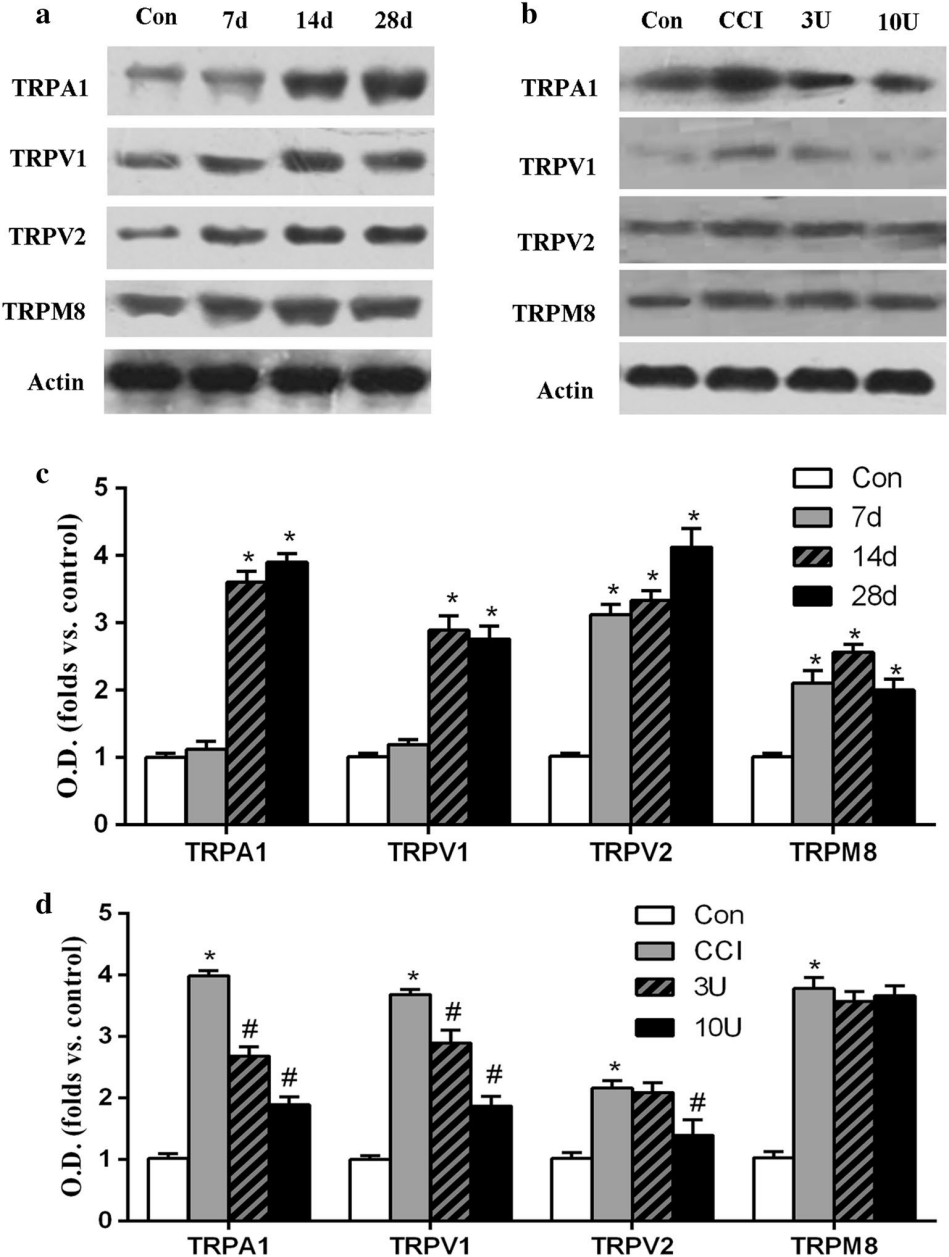
The protein levels of TRPs. **a**, **c** Western blots analysis and quantitative of TRPA1, TRPV1, TRPV2 and TRPM8 at various times after ION-CCI. **b**, **d** Western blots analysis and quantitative of TRPA1, TRPV1, TRPV2 and TRPM8 at 7 days after BoNT-A or normal saline injection (21 days after operation) in 4 treatment groups. β-actin was used as an internal standard. Only the representative Western blots of them are illustrated in this figure. Data were mean ± SD (n = 6/group). TG indicates injection into the trigeminal ganglion; WP indicates injection into the facial whisker pad. *P < 0.05 versus control and ^#^P < 0.05 versus CCI group

To obtain insight into the mechanisms of antinociceptive effects of BoNT-A, the protein expression of TRPA1, TRPV1, TRPV2 and TRPM8 was further examined in ION-CCI model of trigeminal neuralgia. Western blot demonstrated that TRPA1 and TRPV1 expression increased remarkable at 14 days (P < 0.05) after ION-CCI and increased until 28 days (P < 0.05), TRPV2 increased remarkable at 7 days (P < 0.05) after ION-CCI and increased until 28 days (P < 0.05), TRPM8 expression started to increase at 7 days (P < 0.05) after ION-CCI, reached a maximum level at 14 and remained significantly elevated until 28 days (P < 0.05) (Fig. 4a). While subcutaneously administration of BoNT-A (3, 10 U/kg) significantly decreased the expression of TRPA1 and TRPV1 at 7 days after BoNT-A injection in a dose-related manner (P < 0.05). BoNT-A (10 U/kg rather than 3 U/kg) significantly decreased the expression of TRPV2 at 7 days after BoNT-A injection. TRPM8 expression was not change significantly after BoNT-A (3, 10 U/kg) treatment compared with control group (P > 0.05) (Fig. 4b).

## Discussion

The application of BoNT-A has been recently explored in a number of pain associated disorders, such as diabetic neuropathy, complex regional pain syndrome, trigeminal neuralgia, and occipital neuralgia (Oh and Chung 2015). In recent years, a number of clinical studies have shown that BoNT-A treatment for TN is safe and effective (Zuniga et al. 2008; Ngeow and Nair 2010; Wu et al. 2012; Zhang et al. 2014; Li et al. 2014). However, the intrinsic limitations of clinical studies hamper the in-depth analysis on its mechanism. In recent years, researchers have explored the treatment and mechanism of BoNT-A for pain associated with trigeminal nerve region (Matak et al. 2011; Kim et al. 2015). However, these studies essentially use the formalin-induced inflammatory pain model and BoNT-A pretreatment method to study the mechanism. The features of formalin-induced inflammatory pain model are inconsistent with those of TN. In addition, BoNT-A pretreatment method is not a good clinical simulation of BoNT-A treatment for TN. The ION-CCI model is widely accepted as an appropriate model of trigeminal neuralgia (Vos et al. 1994). In this study, we used the ION-CCI model of TN and examined the antinociceptive effects of BoNT-A in successfully generated model, which is a good animal model for studying the clinical BoNT-A treatment for TN.

In this study, we found that BoNT-A significantly increased the mechanical stimulation threshold in rat ION-CCI model of trigeminal neuralgia, which is similar to the results observed in a previous study (Filipovic et al. 2012). However, most previous studies on the ION-CCI model of TN use BoNT-A doses based on the doses used in other pain models. In this study, we found that differences in antinociceptive effects between different doses of BoNT-A in ION-CCI model of TN were not statistically significant, which is similar to the results of our previous clinical studies that there is no statistically significant differences in clinical efficacy between low-dose (25U) and high-dose (75U) of BoNT-A treatment in TN patients (Zhang et al. 2014). This also suggests that the animal model and experimental method used in this study are consistent with the features of clinical BoNT-A treatment for TN.

The treatment mechanism of BoNT-A for TN is currently unclear. Most previous studies suggest that BoNT-A acts locally or on the trigeminal ganglia (Cui et al. 2004; Xiao et al. 2013). Vc is the primary relay for orofacial pain and temperature sensations and the site for processing sensory information, and plays an important role in the mechanism of TN pathogenesis. In this study, we used a specific BoNT-A marker, cSNAP-25, to determine the possible sites of BoNT-A action in the ION-CCI model of TN. By combining colchicine injection to block axonal transport, we proved that BoNT-A exerts antinociceptive effects in brainstem Vc via axonal transport in the ION-CCI model of TN. The results are similar to the BoNT-A study results in other models (Matak et al. 2011, 2012). Matak et al. inject BoNT-A locally in the sciatic nerve area and detect cSNAP-25 in the corresponding spinal cord sections.

Since BoNT-A acts on the central nervous system through axonal transport, we examined whether BoNT-A affects the motor coordination ability in rats. To the best of our knowledge, this study first used Rota-rod test to demonstrate that BoNT-A injection into facial trigeminal nerve region did not cause systemic effects in rats even at high doses (10 U/kg). This suggests that BoNT-A exerts specific antinociceptive function in the central nervous system without affecting its other functions.

In recent years, TRPs have been identified as nonselective cation channel proteins localized in the plasma membrane and membranes of intracellular organelles. A significant difference between TRPs family proteins and other ion channel family proteins is that members of TRP family share low homology and can be activated or sensitized by a variety of mediators and ligands. It is currently recognized that TRPA1, TRPV1, TRPV2 and TRPM8 play an important role in the pathogenesis of pain sensation production and hyperalgesia (Ferrandiz-Huertas et al. 2014), and are involved in the perception of pain induced by chemical, temperature or mechanical stimuli (Mickle et al. 2015). Most previous studies on ION-CCI model of TN focus on pathological changes of trigeminal ganglia. To the best of our knowledge, this study first demonstrated that TRPA1, TRPV1, TRPV2 and TRPM8 expression elevated in the Vc in ION-CCI model of TN, and BoNT-A effectively inhibited the high expression of TRPA1, TRPV1 and TRPV2. This suggests that BoNT-A is able to reduce central sensitization and therefore exerts antinociceptive function by inhibiting the high expression of nociceptors, such as TRPA1, TRPV1 and TRPV2. In addition, we also found that BoNT-A had no effect on the increased expression of TRPM8, hence suggesting BoNT-A does not affect TRPM8 expression. However, the effects of BoNT-A on TRPM8 require further study to confirm.

## Conclusions

In conclusion, our findings suggest that peripherally applied BoNT-A can produce antinociceptive effects in ION-CCI model. The underlying mechanisms may be BoNT-A directly acts on the Vc via axonal transport, inhibits the high expression of TRPA1, TRPV1 and TRPV2, and reduces central sensitization. This study provides not only a theoretical basis for clinical application of BoNT-A for TN treatment and other pain associated disorders, but also a new direction for understanding the antinociceptive mechanism of BoNT-A.
